# Supplementary material for: Massive peatland carbon banks vulnerable to rising temperatures
Source: Nat Commun. 2020 May 12;11:2373. doi: 10.1038/s41467-020-16311-8 (PMC7217827; doi:10.1038/s41467-020-16311-8)
Supplement: Supplementary file 1 — Supplementary Information [file 41467_2020_16311_MOESM1_ESM.pdf]

Supplementary Information for "Massive peatland carbon banks vulnerable to rising temperatures" by Hopple et al.:

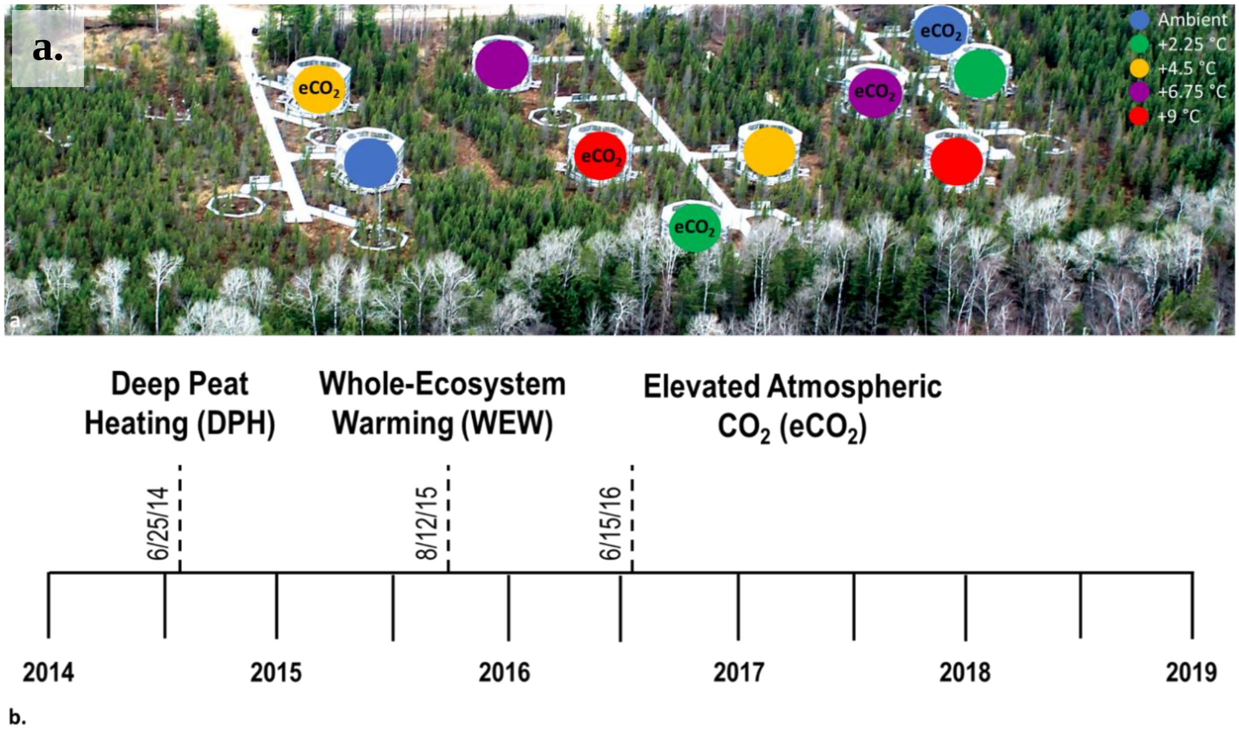

SUPPLEMENTARY FIGURE 1 | **Aerial view of the SPRUCE site located in northern Minnesota and experiment timeline.** Experimental enclosures (**a**) are positioned along three boardwalks that transect an ombrotrophic bog and are subjected to whole-ecosystem warming (WEW) and elevated atmospheric CO_2 concentrations (eCO_2). (**b**) The various treatments were introduced sequentially over time. Colors denote temperature differentials targeted within each enclosure and "eCO_2" identifies those exposed to elevated atmospheric CO_2 concentrations. DPH = deep-peat heating. Ambient temperatures are in blue, +2.25 in green, +4.5 in yellow, +6.75 in purple, and +9 in red.

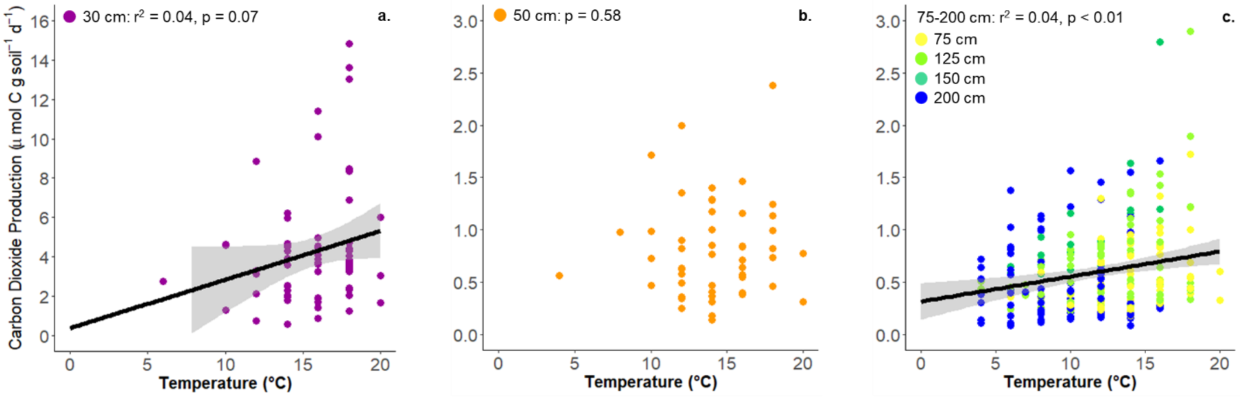

SUPPLEMENTARY FIGURE 2 | **Depth-specific CO_2 production in anaerobic incubations.** Carbon dioxide production temperature responses from peat samples collected (**a**) 30 cm, (**b**) 50 cm, and (**c**) 75-200 cm below the hollow surface and anaerobically incubated within 1°C of in-situ temperatures. Peat samples were collected 1-4 times per year during the growing season and over the course of four years (2015-2018) throughout whole-ecosystem warming. Linear regressions with 95% confidence intervals are shown in black and grey, respectively. Colors represent different depth increments.

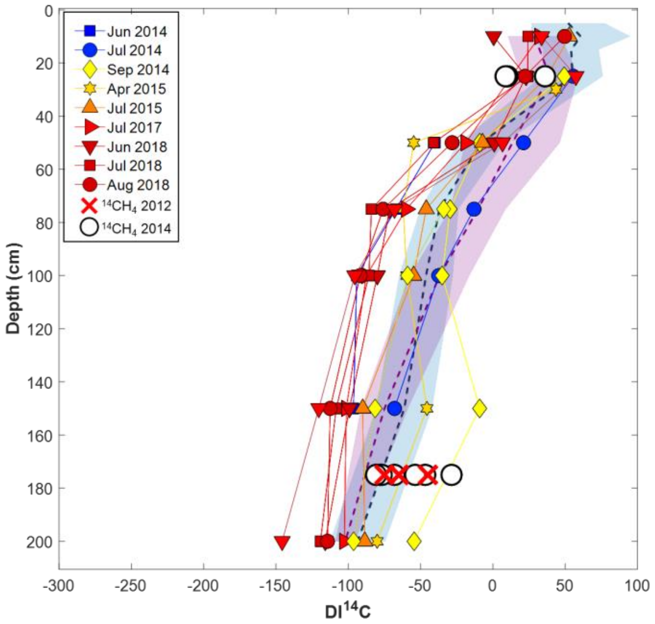

SUPPLEMENTARY FIGURE 3 | **Comparison of CH_4 and DIC radiocarbon signatures.** Depleted $^{14}CH_4$ and DI^{14}C signatures (in ‰) from three of the warmest enclosures (+6.75 °C with ambient CO_2, +9 °C with ambient CO_2, and +9 °C with elevated CO_2). The 2012 $^{14}CH_4$ data (red x's) were collected from the control, as well as the ambient and eCO_2 +9°C plots. The 2014 $^{14}CH_4$ data (open circles) were collected from 25 cm in the ambient CO_2 +6.75°C, elevated CO_2 control and +9°C plots as well as at 175 cm in the ambient CO_2 +0°C enclosure and both the ambient and elevated CO_2 of the +4.5°C, +6.75°C and +9°C enclosures. As in the main Figure 2, the shaded areas and dotted lines indicate the DI^{14}C LOESS locally weighted polynomial regression smooth curves and 95% confidence intervals from one ambient temperature enclosure (pink shading) and one reference plot with no infrastructure (blue shading).

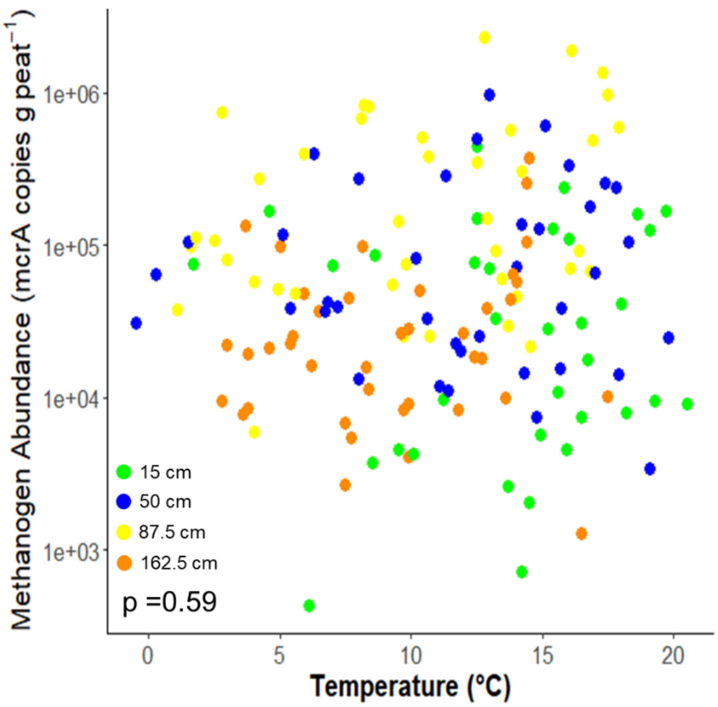

SUPPLEMENTARY FIGURE 4 | **Depth-specific methanogen abundance.** The relationship between temperature and methanogen abundance throughout the soil profile at S1 Bog. Gene abundance was determined by quantitative PCR using primers targeting the *mcrA* gene for methanogens. Microbial abundance is shown for samples collected from enclosures that were +0, +4.5, and +9 °C above ambient temperatures. Cores were collected 1-2 times per year during deep-peat heating (2014) and whole-ecosystem warming (2015-2017). The effect of temperature did not vary by depth ($p = 0.59$) or over time ($p = 0.20$); thus, we present the response of methanogens to temperature across all depths and sampling events. Different depth increments are represented by different colors.

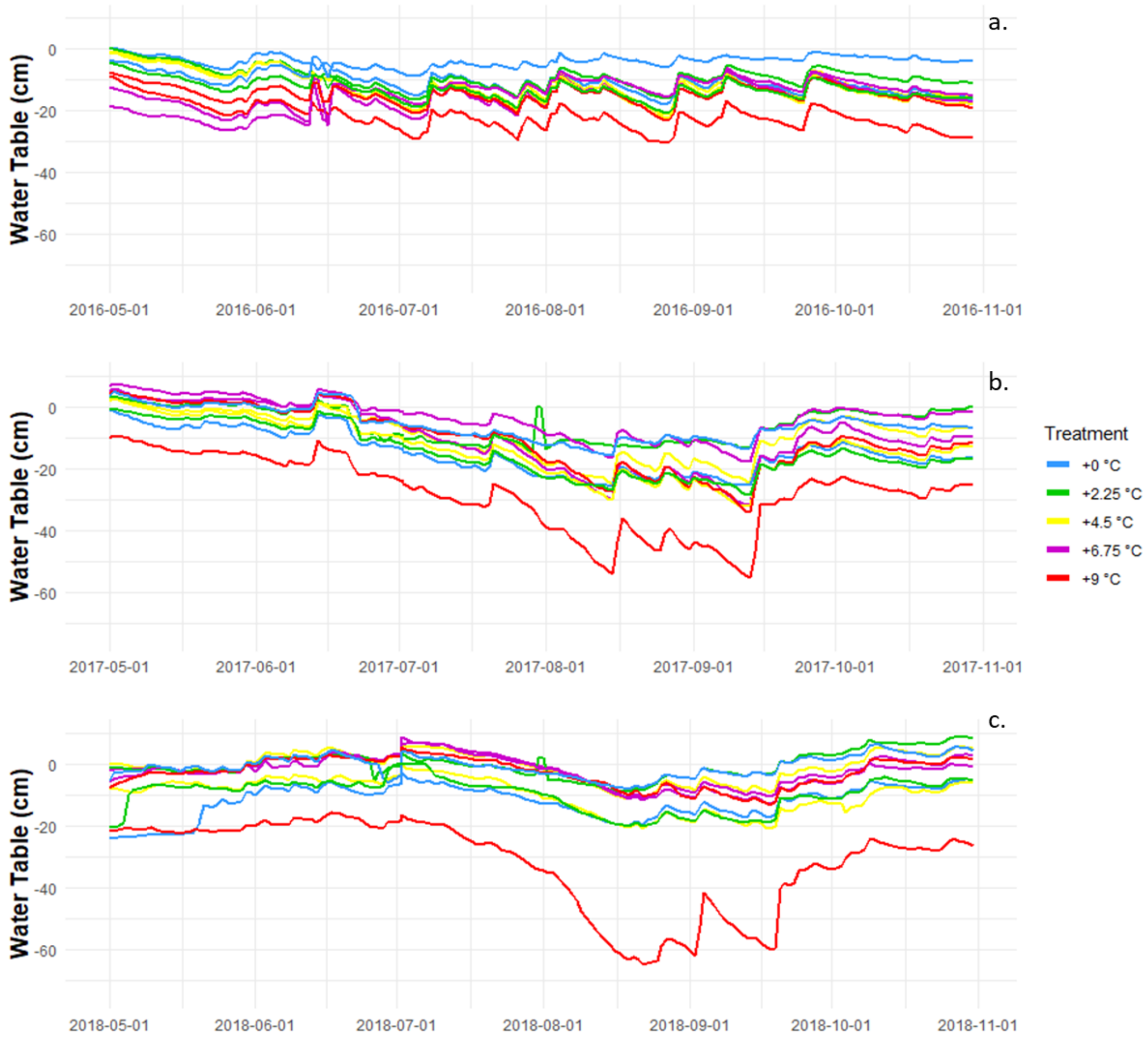

SUPPLEMENTARY FIGURE 5 | **Water-table position at S1 Bog.** Enclosure-specific water position from May through November in **(a)** 2016, **(b)** 2017, and **(c)** 2018. Data were unavailable for 2015 and are unreliable from November through April due to freezing; thus, we only present changes in water-table position during thaw periods. These data demonstrate the annual drawdowns in water-table that can occur during warmer, summer months, as well as those that can occur due to the warming treatments. Colors represent temperature treatments.

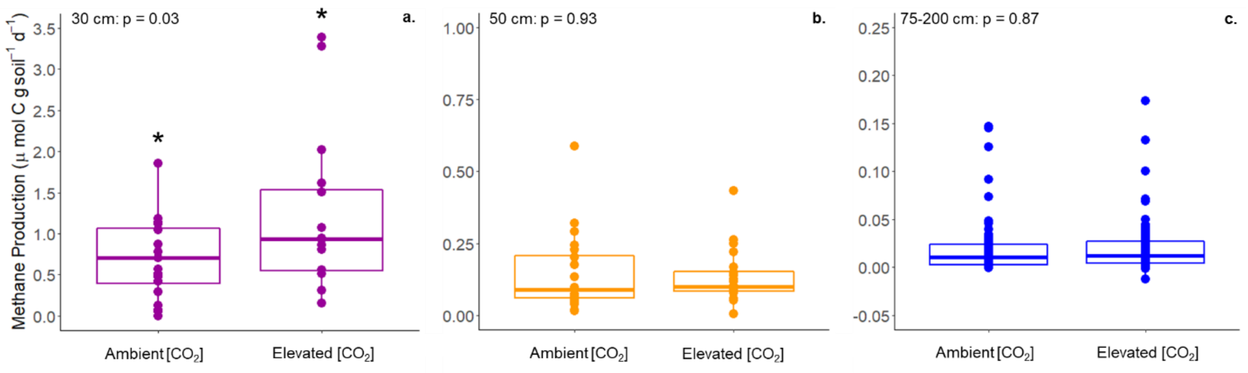

SUPPLEMENTARY FIGURE 6 | **Depth-specific effects of eCO_2 on CH_4 production.** Methane production from (**a**) 30 cm, (**b**) 50 cm, and **(c)** deep (75-200 cm) peat samples that were anaerobically incubated within 1 °C of in situ temperatures following two years of exposure to elevated atmospheric CO_2 concentrations. eCO_2 stimulated rates of CH_4 production only in surficial soil layers (**a**). The thick lines represent the median value, the box edges denote the upper and lower 25% quartiles, and the whiskers show the maximum and minimum values. * = significant difference.

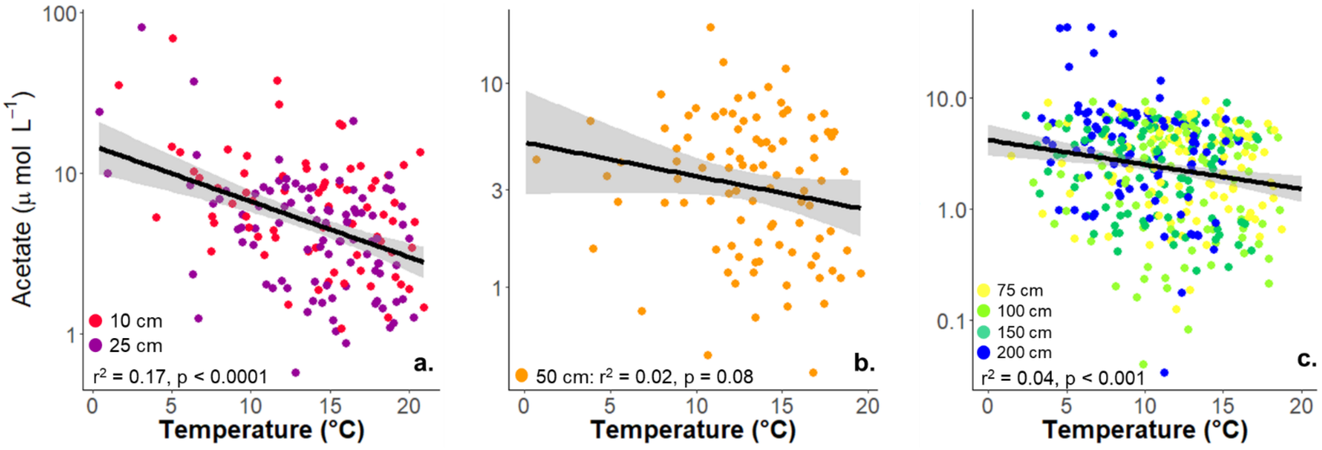

SUPPLEMENTARY FIGURE 7 | **Depth-specific porewater acetate concentrations.** Decreasing temperature responses of porewater acetate concentrations collected from piezometers installed **(a)** 10 and 25 cm, **(b)** 50 cm, and **(c)** 75-200 cm below the hollow surface at S1 Bog. Samples were collected 1-4 times per year during the growing season in 2015, 2016, and 2018. Colors represent different sampling depths. Linear regressions with 95% confidence intervals are shown in black and grey, respectively.

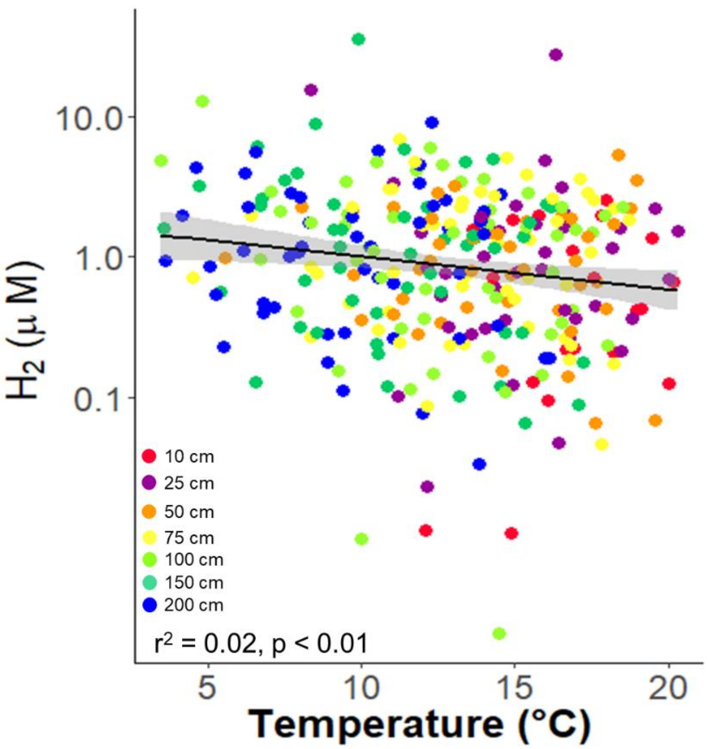

SUPPLEMENTARY FIGURE 8 | **Depth-specific porewater di-hydrogen concentrations.** Decreasing temperature responses of porewater hydrogen concentrations collected from piezometers installed 10-200 cm below the hollow surface at S1 Bog. Samples were collected 4 times per year during the growing season in 2016. Colors represent different sampling depths. The linear regression and 95% confidence intervals are shown in black and grey, respectively.

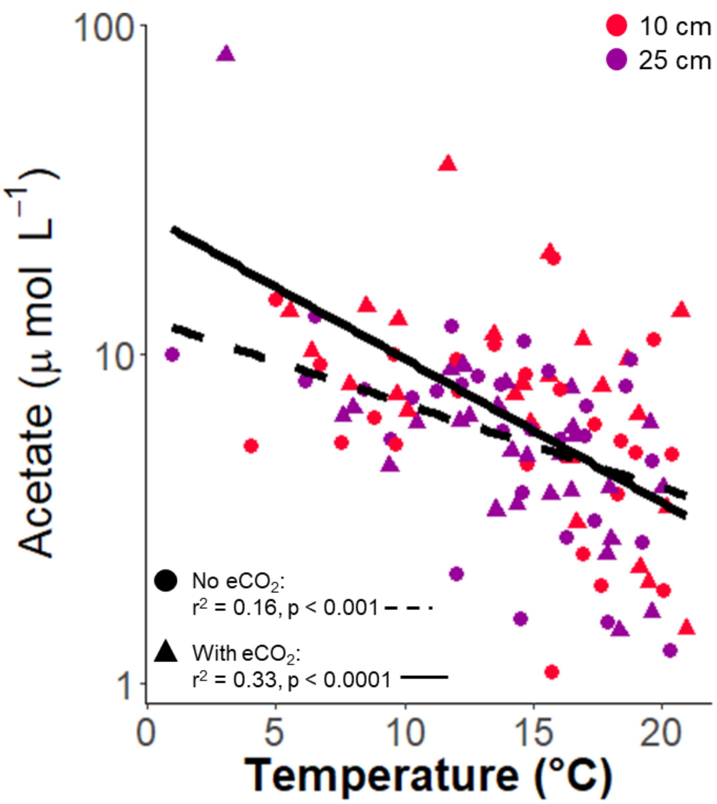

SUPPLEMENTARY FIGURE 9 | **Decreasing temperature responses of porewater acetate concentrations collected from piezometers installed 10 and 25 cm below the hollow surface at S1 Bog.** The strength of the temperature response depended on exposure to elevated atmospheric CO_2 concentrations (eCO_2; $p < 0.05$), with those under eCO_2 exhibiting a steeper response. Samples were collected 1-4 times per year during the growing season in 2016 and 2018. Colors represent different sampling depths. Shapes denote atmospheric CO_2 conditions. Linear regressions with 95% confidence intervals are shown in black and grey, respectively.

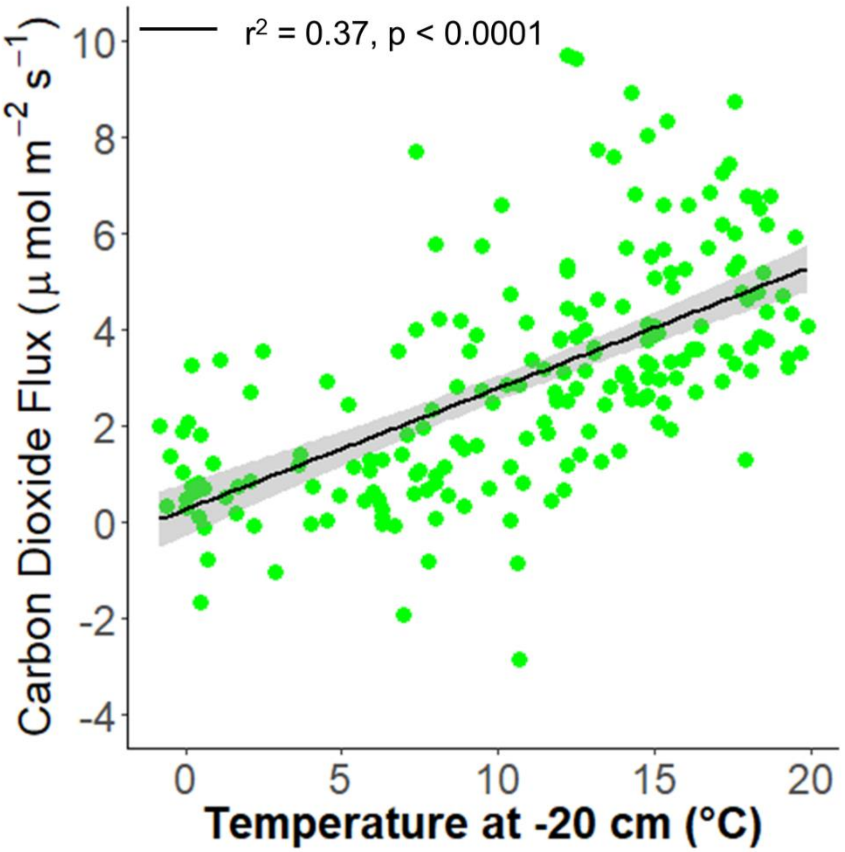

SUPPLEMENTARY FIGURE 10 | **Dark CO_2 flux from S1 Bog.** Positive temperature response of surface CO_2 emissions following five years of warming. Measurements were made 6-8 times per year during 2015-2018. Linear regressions with 95% confidence intervals are shown in black and grey, respectively.

SUPPLEMENTARY TABLE 1 | **Depth-specific contributions to total CH_4 production.** Methane production rates from multiple depth increments (20-30, 40-50, 50-75, 100-125, 125-150, and 175-200 cm) were determined from eight field sampling events (2015-2018) and subsequent anaerobic laboratory incubations of 1:1 peat to porewater mixtures at in-situ temperatures. There empirical data were used to extrapolate CH_4 production rates for non-sampled depths (30-40, 75-100, and 150-175 cm) up to a depth of 2 m. Depth-specific rates of CH_4 production were then averaged across eight sampling events in order to determine the percent of total CH_4 production attributed to individual depth increments. Here, we see that roughly 75% of total CH_4 production is generated within the top 30 cm of the anaerobic zone (20-50 cm below the hollow surface).

**Depth (cm)**	**Average CH_4 production ± standard error (µmol C g peat^{-1} d^{-1})**	**Percent contribution to total CH_4 production (%)**
20-30	0.46 ± 0.05	30
30-40	0.38 ± 0.09	25
40-50	0.25 ± 0.14	17
50-75	0.15 ± 0.02	10
75-100	0.10 ± 0.01	7
100-125	0.06 ± 0.01	4
125-150	0.05 ± 0.01	3
150-175	0.03 ± <0.01	2
175-200	0.03 ± <0.01	2
